# White Matter Hyperintensities and Mild TBI in Post-9/11 Veterans and Service Members

**DOI:** 10.1093/milmed/usae336

**Published:** 2024-07-13

**Authors:** David F Tate, Erin D Bigler, Gerald E York, Mary R Newsome, Brian A Taylor, Andrew R Mayer, Mary Jo Pugh, Angela P Presson, Zhining Ou, Elizabeth S Hovenden, Josephine Dimanche, Tracy J Abildskov, Rajan Agarwal, Heather G Belanger, Aaron M Betts, Timothy Duncan, Blessen C Eapen, Carlos A Jaramillo, Michael Lennon, Jennifer E Nathan, Randall S Scheibel, Matthew B Spruiell, William C Walker, Elisabeth A Wilde

**Affiliations:** TBI and Concussion Center, Department of Neurology, University of Utah School of Medicine, Salt Lake City, UT 84103, USA; George E. Wahlen Veterans Affairs Medical Center, Salt Lake City, UT 84103, USA; Departments of Psychology and Neuroscience, Brigham Young University, Provo, UT 84604, USA; TBI and Concussion Center, Department of Neurology, University of Utah School of Medicine, Salt Lake City, UT 84103, USA; Departments of Psychology and Neuroscience, Brigham Young University, Provo, UT 84604, USA; Alaska Radiology Associates, Anchorage, AK 99508, USA; Departments of Neurology and Psychiatry, University of New Mexico, Albuquerque, NM 87131, USA; Michael E. De Bakey Veterans Affairs Medical Center, Houston, TX 77030, USA; Department of Physical Medicine and Rehabilitation, Baylor College of Medicine, Houston, TX 77030, USA; Department of Imaging Physics, The University of Texas MD Anderson Cancer Center, Houston, TX 77030, USA; Departments of Neurology and Psychiatry, University of New Mexico, Albuquerque, NM 87131, USA; George E. Wahlen Veterans Affairs Medical Center, Salt Lake City, UT 84103, USA; Department of Internal Medicine, University of Utah School of Medicine, Salt Lake City, UT 84103, USA; Department of Internal Medicine, University of Utah School of Medicine, Salt Lake City, UT 84103, USA; Department of Internal Medicine, University of Utah School of Medicine, Salt Lake City, UT 84103, USA; TBI and Concussion Center, Department of Neurology, University of Utah School of Medicine, Salt Lake City, UT 84103, USA; TBI and Concussion Center, Department of Neurology, University of Utah School of Medicine, Salt Lake City, UT 84103, USA; TBI and Concussion Center, Department of Neurology, University of Utah School of Medicine, Salt Lake City, UT 84103, USA; Departments of Psychology and Neuroscience, Brigham Young University, Provo, UT 84604, USA; Michael E. De Bakey Veterans Affairs Medical Center, Houston, TX 77030, USA; Defense and Veterans Brain Injury Center (DVBIC), MacDill AFB, FL 33621, USA; Department of Radiology, Brooke Army Medical Center, San Antonio, TX 78234, USA; Portland Veterans Hospital, Portland, OR 97239, USA; VA Greater Los Angeles Health Care System, Los Angeles, CA 90073, USA; South Texas Veterans Health Care System, San Antonio, TX 78229, USA; TBI and Concussion Center, Department of Neurology, University of Utah School of Medicine, Salt Lake City, UT 84103, USA; Department of Radiology, Johns Hopkins Medical School, Baltimore, MD 21205, USA; Michael E. De Bakey Veterans Affairs Medical Center, Houston, TX 77030, USA; Department of Physical Medicine and Rehabilitation, Baylor College of Medicine, Houston, TX 77030, USA; TBI and Concussion Center, Department of Neurology, University of Utah School of Medicine, Salt Lake City, UT 84103, USA; Department of Physical Medicine and Rehabilitation, Baylor College of Medicine, Houston, TX 77030, USA; Department of Physical Medicine and Rehabilitation, Virginia Commonwealth University, Richmond, VA 23220, USA; Richmond Veterans Affairs (VA) Medical Center, Central Virginia VA Health Care System, Richmond, VA 23249, USA; TBI and Concussion Center, Department of Neurology, University of Utah School of Medicine, Salt Lake City, UT 84103, USA; George E. Wahlen Veterans Affairs Medical Center, Salt Lake City, UT 84103, USA; Department of Physical Medicine and Rehabilitation, Baylor College of Medicine, Houston, TX 77030, USA

## Abstract

**Introduction:**

The neurobehavioral significance of white matter hyperintensities (WMHs) seen on magnetic resonance imaging after traumatic brain injury (TBI) remains unclear, especially in Veterans and Service Members with a history of mild TBI (mTBI). In this study, we investigate the relation between WMH, mTBI, age, and cognitive performance in a large multisite cohort from the Long-term Impact of Military-relevant Brain Injury Consortium—Chronic Effects of Neurotrauma Consortium.

**Materials and Methods:**

The neuroimaging and neurobehavioral assessments for 1,011 combat-exposed, post-9/11 Veterans and Service Members (age range 22-69 years), including those with a history of at least 1 mTBI (*n* = 813; median postinjury interval of 8 years) or negative mTBI history (*n* = 198), were examined.

**Results:**

White matter hyperintensities were present in both mTBI and comparison groups at similar rates (39% and 37%, respectively). There was an age-by-diagnostic group interaction, such that older Veterans and Service Members with a history of mTBI demonstrated a significant increase in the number of WMHs present compared to those without a history of mTBI. Additional associations between an increase in the number of WMHs and service-connected disability, insulin-like growth factor-1 levels, and worse performance on tests of episodic memory and executive functioning-processing speed were found.

**Conclusions:**

Subtle but important clinical relationships are identified when larger samples of mTBI participants are used to examine the relationship between history of head injury and radiological findings. Future studies should use follow-up magnetic resonance imaging and longitudinal neurobehavioral assessments to evaluate the long-term implications of WMHs following mTBI.

## INTRODUCTION

Without additional imaging or clinical findings, white matter hyperintensities (WMHs) identified on magnetic resonance imaging (MRI) are often considered “nonspecific” findings by neuroradiologists. These lesions are common in the general population older than 50 years and are typically associated with vascular risk factors, including hypertension.^1^ White matter hyperintensities may also be associated with expanded perivascular spaces, small cerebral vessel disease, and systemic inflammation.^2,3^ Importantly, there is evidence that WMHs are predictive of poor clinical outcomes (i.e., stroke, dementia, and death^4,5^) in older patient populations as well as poor functional and clinical outcomes in the general population (i.e., poor sleep quality). Consequently, WMHs should be considered clinically relevant and should prompt additional screening for other risk factors when present. Historically, periventricular and deep white matter regions have been considered the most frequent location of nonspecific WMHs. However, this finding is based on adults predominantly older than 50 years, and the functional consequences of these imaging findings are still being investigated.^1^ Evidence shows that WMHs are related to cognitive function in prospective research approaches,^6^ and in meta-analytic approaches, progression in size and number of WMHs can be associated with cognitive decline (Lange, 2014 #546).^1^

The question of whether WHMs can result from traumatic brain injury (TBI) is also highly important and concerning for more than 445,000 US military personnel who sustained a TBI since the beginning of the Iraq and Afghanistan conflicts. In 1 study of 834 Service Members, WMHs in those who had sustained a TBI were examined.^7^ This sample included Service Members who were 3.8 years postinjury and included a significant number of participants with a mild TBI (mTBI; 768 or 92% of the sample) and blast-related mechanism of injury (84% of the sample). One or more WMHs were present in 432/834 (51.8%) of the TBI group compared with 38.1% of uninjured controls (mean age = 31 years). Compared to similarly aged, uninjured Service Members (1/42 or 2.4%), a higher proportion of the mTBI group (187/834 or 22.4%) had more WMHs than expected.^7^ These findings suggest a link between WMH and mTBI that is not well understood.

Other studies have demonstrated an assoication between WMHs and cognitive deficits in combat-exposed Veterans.^8,9^ For example, Clark et al. acquired imaging data for 68 post-9/11 Veterans, including 46 mTBI (mean age = 31 years) and 22 military controls (mean age = 34 years) at least 1 year past their most recent deployment at the time of MRI.^10^ Although WMH volume did not differ between the groups, the presence of a lesion in deep white matter was related to poor delayed recall in the mTBI group, but not in the comparison group after adjusting for post-traumatic stress disorder (PTSD) symptom severity. This dissociation in the relation of WMHs to cognition was corroborated in a recent study which found that in post-9/11 active duty Service Members with mTBI (*N* = 77, mean age = 33 years), the subgroup with WMHs (mean age = 34 years) demonstrated worse working memory compared to those without lesions.^11^

To extend the findings from these studies, we undertook a study of WMHs and their association with mTBI in Service Members and Veterans using data from the ongoing Long-term Impact of Military-relevant Brain Injury Consortium—Chronic Effects of Neurotrauma Consortium (LIMBIC-CENC) prospective longitudinal study. This multisite investigation of combat-exposed Service Members and Veterans includes both those who sustained at least 1 mTBI and those with a negative lifetime history of mTBI.^3^ In contrast to most civilian studies of WMHs, this sample included individuals predominantly younger than 50 years at enrollment. Among this large multisite sample, we analyzed the association of WMHs with mTBI and the relation of WMHs to demographic features, injury variables, medical comorbidities, and clinical and cognitive outcome measures. We hypothesized that WMHs would be found more frequently in the mTBI cohorts and they would relate to cognitive dysfunction.

## MATERIALS AND METHODS

### Participants

As of September 2016, 1,550 LIMBIC/CENC Service Members and Veterans enrolled in the prospective longitudinal study. After excluding participants who were pregnant, had effort validity failure on the medical symptom validity test, had an unknown WMH status, or missing the number of WMHs, there were 1,011 participants. Inclusion in this study required a standardized review of the participant’s MRI by a board-certified neuroradiologist. The sample included 813 Service Members and Veterans who had sustained at least 1 mTBI and 198 with a completely negative lifetime history of mTBI. The inclusion criteria were as follows: (1) age ≥18 years; (2) history of at least 1 combat deployment; (3) combat exposure (score of >1 on at least 1 item of the Deployment Risk and Resilience Inventory-2).^12^ Individuals with severe psychiatric (e.g., schizophrenia) and neurological (e.g., major stroke) disorders were not enrolled. Each participant underwent a detailed semi-structured interview (potential concussive event mapping) to identify all lifetime potential concussive events. Each event was then subjected to a validated and highly standardized mTBI diagnostic interview, described in detail elsewhere, to both validate the diagnosis and allow for the quantification of lifetime exposure, severity of exposure, and context of exposure.^13^ Magnetic resonance imaging and clinical data were selected from the 8 enrollment sites that acquired imaging since the start of the original CENC project.

### Procedures

Each participant underwent an extensive cognitive, symptom, medical, and imaging assessment.^3^ The following is only a brief description of the specific measures included in the analyses conducted for this study.

#### Demographic variables

Demographic variables included in the analyses were age, sex, education level, race, branch of service, and time since index (index event) injury. Of note, many of these demographic factors are included in these analyses as a method of improving the consistency of reporting results in this cohort across research studies, so that more direct comparisons can be made between already published results.

#### Cognitive measures

In this study, a subset of the entire cognitive battery of tests was included in the analyses. For this study, a verbal list learning memory task (California Verbal Learning Test-II), a measure of executive function (inhibition/switching) under time conditions (Trail Making B test), a measure of processing speed (NIH Toolbox Pattern Comparison), executive function (NIH Toolbox Flanker Inhibitory Control), and visual memory (NIH Toolbox Picture Sequence Memory) were assessed. These measures were chosen because they tap into cognitive functions often associated with measures of white matter integrity including WMHs.^14^

#### Medical comorbidities and other measures

In addition to the cognitive outcome measures, cardiovascular health, alcohol use, mental health, and other medical measures were also examined as possible factors associated with WMH burden. Measurements of physical and cardiovascular health included systolic and diastolic blood pressure measures, body mass index (BMI), current smoking status (every day, some days, not at all, unknown), thyroid-stimulating hormone (TSH) levels, and serum insulin-like growth factor-1 (IGF-1). These variables were selected and included in the analyses given their association with white matter changes or WMH following TBI, in general patient populations, or in other neurologic conditions.^15,16^

Symptom inventories used to assess various comorbid conditions commonly observed in Veteran/military cohorts were also included in the analyses. The battery included measures for PTSD (Mini International Neuropsychiatric Interview [MINI]), hazardous use of alcohol (Alcohol Use Disorders Identification Test [AUDIT-C]), and depression symptom severity (Patient Health Questionnaire [PHQ-9]).

#### Magnetic resonance imaging

**Figure 1.**
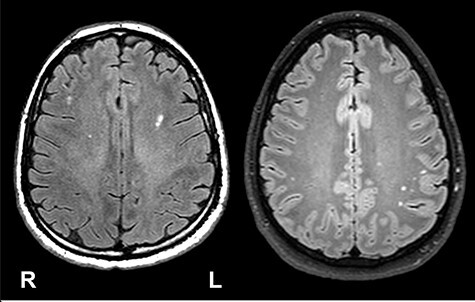
Examples of WMH visable in this cohort.

The imaging protocol for LIMBIC-CENC included a set of 3D, high-resolution T1-weighted, T2-weighted, and T2 fluid-attenuated inversion recovery (FLAIR) images. The LIMBIC-CENC Imaging Core (University of Utah) monitored the quality of MRI at all sites, including ensuring that the prescribed sequence parameters for the scans remain consistent within and across sites. The neuroradiologists participated in a training workshop and regular conference calls to reach a consensus on their approach to reading the scans and quantifying various aspects of WMH present. The FLAIR images were used to identify WMHs for each participant. The other image sequences were utilized to verify findings only when there are concerns or questions about the findings observed in the FLAIR imaging. Inter-rater reliability over a 1-month interval for the participating neuroradiologists was excellent (*r* > 0.95). Clinical reads were performed without access to the group classification of the participant. Figure 1 shows examples of periventricular WMHs on an axial FLAIR image.

For this report, WMH presence (yes/no) and the number of WMHs expressed in ordinal categories (i.e., 0 WMHs, 1-2 WMHs, 3-5 WMHs, 6-10 WMHs, 11-20 WMHs, and >20 WMHs) were used. These categories were arbitrarily decided by our group after looking at the distribution of WMHs counts across the sample and trying to preserve statistically similar sample sizes. Counts were investigated in this study for 2 reasons. First, count should be considered more clinically relevant in the neuroradiology setting, where counts are more readily available compared to volumes or other types of methods intended to quantify WMHs. Second, this study aims to identify potentially relevant clinical and cognitive variables that would be investigated using more rigorous quantitative methods available.

### Statistical Analysis

We summarized continuous demographics and clinical outcomes of interest using mean and standard deviation^17^ or median and interquartile range and categorical variables using counts and percentages. We compared these variables between the 2 groups, defined by the presence or absence of mTBI, using nonparametric Wilcoxon rank-sum tests for skewed continuous variables and chi-squared tests for categorical variables. We conducted univariable regression analyses for both binary WMH and ordinal WMH outcomes. We then constructed a multivariable model for our binary WMH outcome by performing stepwise variable selection using the Akaike Information Criterion. In addition to mTBI status, which was forced in to the model selection since it was our primary predictor of interest, candidate variables for our multivariable model included the 30 demographic and clinical variables of interest presented in **Table 1**, as well as: MINI: Current PTSD Diagnosis, BMI, DRRI-2-Combat Completion code, Ethnicity, currently in the military, California Verbal Learning Test, second edition (CVLT-II): Total Raw Score, CVLT-II: Short Delay Cued Recall Raw Score, CVLT-II: Long Delay Cued Recall Raw Score, and their two-way interactions with mTBI. We reported results as odds ratios (ORs) with 95% CIs and *P*-values. The model that was selected for our binary WMH outcome was then refit to the ordinal WMH outcome using a proportional odds cumulative logit regression model. We reported results as proportional ORs with their 95% CIs and *P*-values. Proportional odds are interpreted as the odds of increment, which are consistent for any increase in WMH (i.e., the odds of moving from 0 WMH to a higher WMH category are the same as the odds of moving from 20 or fewer WMH to >20 WMH). Since we repeated the same analysis on both binary and ordinal versions of the WMH outcome, but mTBI was our primary predictor of interest in both cases, we used a Bonferroni-adjusted significance level of 0.025 (=0.05/2) for assessing statistical significance to control the Type I error. Statistical analyses were implemented using R v. 4.0.3 (R Core Team, 2020).

**Table 1.** Variables of Interest by Group

Variable	No mTBI (*N* = 198)	mTBI (*N* = 813)	*P*-value	No MRI (*N* = 468)	MRI (*N* = 1082)	*P*-value
WMH binary: present	73 (37%)	315 (39%)	.63^a^	–	–	–
Sex: female	43 (21.7%)	94 (11.6%)	**<.001^a^**	58(12.4%)	145(13.4%)	.59^a^
Age at baseline: mean (SD)	40.3 (10.4)	40.0 (9.7)	.96^b^	39.7 (9.4)	40.0 (9.8)	.73^b^
Education: 12 years or less	27 (13.6%)	114 (14%)	.87^a^	78 (16.7%)	148 (13.7%)	.10^a^
College 1 year to 3 years	76 (38.4%)	326 (40.1%)	–	202 (43.2%)	441 (40.8%)	–
College 4 years or more	95 (48%)	373 (45.9%)	–	188 (40.2%)	493 (45.6%)	–
Race: White	143 (72.2%)	603 (74.2%)	.24^a^	315 (67.3%)	795 (73.5%)	**.019^a^**
Time from index date to consent date (years): mean (SD)	9.9 (5.2)	9.7 (4.7)	.90^b^	10.1 (4.9)	9.8 (4.8)	.09^b^
Branch of service: Army	124 (62.6%)	529 (65.2%)	**.009^a^**	325 (70%)	701 (64.9%)	.25^a^
Air Force	27 (13.6%)	89 (11%)	–	40 (8.6%)	119 (11%)	–
Navy	27 (13.6%)	62 (7.6%)	–	37 (8%)	96 (8.9%)	–
Reserves	20 (10.1%)	131 (16.2%)	–	62 (13.4%)	164 (15.2%)	–
PHQ-9: major depressive disorder criteria met: no	174 (87.9%)	654 (80.7%)	**.019^a^**	352 (76.5%)	877 (81.4%)	.030^a^
AUDIT-C: level of consumption hazardous? No	141 (71.2%)	506 (62.6%)	**.024^a^**	307 (66.3%)	692 (64.3%)	.45^a^
Do you have a service-connected disability? Yes	134 (67.7%)	564 (69.4%)	.64^a^	319(68.2%)	752(69.5%)	.60^a^
Systolic: mean (SD)	125.0 (12.3)	125.9 (13.8)	.47^b^	127.4 (15.2)	125.9 (13.7)	.17^b^
Diastolic: mean (SD)	80.6 (10.0)	80.9 (10.0)	.91^b^	81.4 (10.4)	80.9 (10.0)	.63^b^
Do you now smoke: every day	25 (12.6%)	90 (11.1%)	.45^a^	77 (16.5%)	124 (11.5%)	**.011^a^**
Some days	8 (4%)	53 (6.5%)	–	39 (8.3%)	69 (6.4%)	–
Not at all	68 (34.3%)	253 (31.1%)	–	126 (26.9%)	346 (32%)	–
Unknown	97 (49%)	417 (51.3%)	–	226 (48.3%)	543 (50.2%)	–
Weight (lbs): mean (SD)	209.3 (38.3)	204.3 (41.5)	.045^b^	206.7 (41.8)	205.8 (41.1)	.45^b^
TSH result: (mIU/L): mean (SD)	1.9 (2.1)	1.8 (1.4)	.94^b^	1.8 (1.1)	1.8 (1.5)	.60^b^
IGF-1 result: (ng/mL): Mean (SD)	166.3 (58.4)	160.7 (53.1)	.34^b^	154.4 (48.7)	161.3 (53.9)	.06^b^
CVLT-II: Short Delay Free Recall Raw Score: mean (SD)	10.4 (3.2)	10.3 (3.3)	.66^b^	9.8 (3.3)	10.2 (3.3)	.040^b^
CVLT-II: Long Delay Free Recall Raw Score: mean (SD)	10.5 (3.3)	10.6 (3.4)	.95^b^	9.8 (3.5)	10.4 (3.4)	**.004^b^**
List Sort Working Memory Unadjusted Score: mean (SD)	103.3 (19.9)	100.8 (18.3)	.14^b^	100.2 (18.7)	100.9 (18.8)	.63^b^
Trail Making B score: mean (SD)	60.8 (22.2)	64.7 (25.1)	.11^b^	71.8 (30.0)	65.8 (26.8)	**<.001^b^**
Pattern Comparison Raw Score: mean (SD)	49.3 (12.8)	48.4 (12.1)	.55^b^	47.8 (12.2)	48.3 (12.4)	.46^b^
Flanker Inhibitory Control Computed Score: mean (SD)	7.9 (7.8)	6.9 (12.1)	.07^b^	7.6 (7.2)	7.2 (11.0)	**.014^b^**
Picture Sequence Memory Raw Score: mean (SD)	104.7 (15.7)	102.0 (14.0)	**.025^b^**	100.1 (14.9)	102.0 (14.4)	**.015^b^**
GPB Nondominant hand time (sec): mean (SD)	75.0 (14.4)	77.4 (15.5)	.043^b^	81.4 (20.4)	77.6 (16.2)	**.004^b^**

Abbreviation: GPB = Grooved Pegboard.

Bold values indicate results significant at the *P* < .025 level.

Number of missing values (mTBI groups): Branch of service: 0/2, PHQ-9: Major Depressive Disorder Criteria Met: 0/3, AUDIT-C: Level of Consumption Hazardous?: 0/5, Systolic = 1/2, Diastolic = 1/2, Weight (lbs) = 0/2, TSH Result: (mIU/L) = 22/79, IGF-1 Result: (ng/mL) = 23/86, CVLT-II: Short Delay Free Recall Raw Score = 1/5, CVLT-II: Long Delay Free Recall Raw Score = 1/5, Trail Making B score = 1/3, Pattern Comparison Raw Score = 7/35, Flanker Inhibitory Control Computed Score = 5/25, Picture Sequence Memory Raw Score = 8/38, GPB nondominant hand time (seconds) = 1/2.

Number of missing values (MRI groups): Branch of service: 4/2, PHQ-9: Major Depressive Disorder Criteria Met: 8/4, AUDIT-C: Level of Consumption Hazardous?: 5/6, Systolic = 2/3, Diastolic = 2/3, Weight (lbs) = 3/2, TSH Result: (mIU/L) = 77/107, IGF-1 Result: (ng/mL) = 81/115, CVLT-II: Short Delay Free Recall Raw Score = 4/6, CVLT-II: Long Delay Free Recall Raw Score = 4/6, Trail Making B score = 1/4, Pattern Comparison Raw Score = 26/46, Flanker Inhibitory Control Computed Score = 22/34, Picture Sequence Memory Raw Score = 30/50, GPB nondominant hand time (seconds) = 0/5.

a The chi-squared test.

b Kruskal–Wallis test.

## RESULTS

### Demographics

**Table 1** compares the 2 groups on several demographic and service-related measures. Approximately 88.4% (*n* = 719) of the subjects with mTBI are male, compared to 78.3% (*n* = 155) in the negative mTBI group. When comparing the 2 groups (mTBI vs. no mTBI), a significant difference was noted for gender (*P* < .001), with the no mTBI history group having more females than the mTBI group (21.7% vs. 11.6%, respectively). The mean age for both groups was 40 years old (SD = 10.4 and 9.7; no mTBI and mTBI, respectively). Other demographic information, including education, race, ethnicity, and branch of service, are provided in **Table 1**. There were no significant differences for any other demographic variables.

### Medical, Clinical, and Cognitive Variable Group Differences

The summary statistics for both medical and clinical variables for mTBI and non-mTBI groups are shown in **Table 2**. When compared across TBI groups, BMI, TSH, IGF-1, smoking history, and blood pressure measures were all nonsignificant variables. Current PTSD diagnosis (MINI, 87.3% vs. 73.8%), depression symptom severity (PHQ-9; 87.9% vs. 80.7%), and endorsement of hazardous alcohol use (AUDIT-C; 71.2% vs. 62.6%) were all statistically higher in the mTBI history group compared to the no mTBI group (*P* = .001, .019, and .024, respectively) but otherwise remained elevated in both groups.

**Table 2.** Odds ratio and proportional OR from univariable logistic and ordinal regressions for outcomes WMHs presence/absence and number of WMH, respectively

Variable	Levels	OR (95% CI)	*P*-value*^a^*	Proportional OR (95% CI)	*P*-value*^a^*
mTBI	Yes	1.08 (0.79, 1.5)	.63	1.02 (0.75, 1.39)	.92
Sex	Male	0.7 (0.48, 1)	.050	0.68 (0.49, 0.97)	.031
Age at baseline (years)		1.07 (1.05, 1.08)	**<.001**	1.07 (1.05, 1.08)	**<.001**
10 × time from index date to consent date (years)		1.39 (1.07, 1.81)	**.016**	1.44 (1.11, 1.86)	**.006**
PHQ-9: Major Depressive Disorder Criteria Met	Yes or possible	1.21 (0.87, 1.67)	.26	1.18 (0.86, 1.60)	.31
AUDIT-C: Level of Consumption Hazardous?	Yes	0.83 (0.63, 1.08)	.16	0.83 (0.64, 1.07)	.15
Do you have a service-connected disability?	No or No response/don’t know/not sure	0.74 (0.56,0.98)	.035	0.74 (0.56, 0.96)	.026
Systolic		1 (0.99, 1.01)	.67	1.00 (0.99, 1.01)	.52
Diastolic		1 (0.98, 1.01)	.48	1.00 (0.98, 1.01)	.47
Weight (lbs)		0.99 (0.96, 1.03)	.72	1.00 (1.00, 1.00)	.68
TSH result (mIU/L)		0.93 (0.83, 1.03)	.20	0.94 (0.84, 1.03)	.23
10 × IGF-1 result (ng/mL)		0.97 (0.94, 0.99)	**.014**	0.96 (0.94, 0.99)	**.002**
CVLT-II: Total Raw Score		0.99 (0.97, 1)	.032	0.98 (0.97, 0.99)	**.005**
CVLT-II: Short Delay Free Recall Raw Score		0.96 (0.92, 1)	.027	0.95 (0.92, 0.99)	**.008**
CVLT-II: Long Delay Free Recall Raw Score		0.97 (0.93, 1)	.07	0.96 (0.92, 0.99)	**.019**
List Sort Working Memory Unadjusted Score		0.99 (0.98, 0.99)	**.002**	0.98 (0.98, 0.99)	**<.001**
10 × Trail Making B Score (time to completion)		1.12 (1.06, 1.18)	**<.001**	1.11 (1.06, 1.16)	**<.001**
Pattern Comparison Raw Score		0.97 (0.96, 0.98)	**<.001**	0.97 (0.96, 0.98)	**<.001**
Flanker Inhibitory Control Computed Score		1 (0.99, 1.02)	.69	1.00 (0.99, 1.02)	.56
Picture Sequence Memory Raw Score		0.99 (0.98, 0.99)	**.002**	0.98 (0.98, 0.99)	**<.001**
GPB Nondominant hand time (seconds)		1.02 (1.01, 1.03)	**<.001**	1.02 (1.01, 1.03)	**<.001**
Do you now smoke	2 Some days	1.24 (0.64, 2.38)	.52	1.30 (0.68, 2.46)	.41
	3 Not at all	1.11 (0.71, 1.77)	.65	1.13 (0.73, 1.78)	.59
	Unknown	1.67 (1.09, 2.59)	**.020**	1.74 (1.16, 2.68)	**.009**
	Every day	(Reference)	(Reference)	(Reference)	(Reference)

Abbreviation: GPB = Grooved Pegboard.

a Bold values indicate significance at a Bonferroni-adjusted *P* < .025 significance level.

Performance on the Grooved Pegboard (75.0 ± 14.4 seconds vs. 77.4 ± 15.5 seconds; *P* = .043) and the picture sequence test (NIH Toolbox; 104.7 ± 15.7 vs. 102.0 ± 14.0; *P* = .025) was significantly different, with the mTBI group performing below that of the no mTBI history group. Nonetheless, scores for these 2 tests remained in the “normal” or “average” range of function. No other significant differences were noted for CVLT-II, Trails B time to completion, Pattern Comparison (NIH Toolbox), or the Flanker Inhibitory Control computed scores (NIH Toolbox).

### Association of mTBI with WMHs

Among the 813 subjects in the mTBI group, 39% had at least 1 WMH, while among the 198 without mTBI, 37% had at least 1 WMH (see **Table 2**). The observed difference in WMHs prevalence was not statistically significant between the 2 primary diagnostic groups (*P*-value = .63).

**Figure 2.**
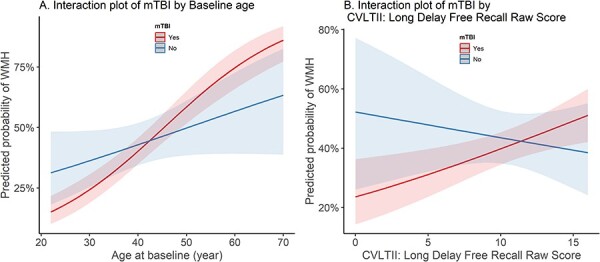
Plots showing how age and CVLTII scores interact with TBI exposure to predict WMH.

The next level of analysis considers the effect of mTBI and other variables on the binary and ordinal WMH outcomes via univariable models (**Table 2**). Results were similar for both outcomes, with several of the neurocognitive measures having statistically significant associations with WMH. Age was also significantly associated with WMH, where a 1-year increase in age was associated with a 7% increase in the odds of WMH (OR = 1.07, 95% CI: 1.05 to 1.08, *P*-value < .001). Supplementary Table S1 shows the results of the selected multivariable model after stepwise variable selection for the binary outcome, as well as the same model fit to the ordinal WMH outcome, using 844 observations out of 1,011. The slope for age was 5% steeper in the mTBI group than the non-mTBI group (OR = 1.05, 95% CI: 1.01 to 1.09, *P*-value = .016, Supplementary Table S1). However, the interaction mTBI × CVLT-II: Long Delay Free Recall Raw Score was not significant (*P*-value = .06). The 2 selected interaction terms were plotted in Figure 2A and B, where Figure 2A shows that the prevalence of WMHs tend to increase as baseline age increases for both subjects with and without mTBI. Figure 2B shows that WMHs increase with increasing CVLT-II in mTBI, but WMHs decrease with increasing CVLT-II in the non-mTBI; however, this interaction was not significant in our multivariable model.

### Association of WMHs with Other Medical, Clinical, and Cognitive Variables

The odds of a participant moving from a lower to a higher category of WMH were 4% lower (OR = 0.96, 95% CI: 0.95 to 0.99, *P*-value = .012) for every 10 ng/mL increase in IGF-1 (**Table 2**). Notably, other clinical variables such as hypertension (both diastolic and systolic values), TSH levels, and smoking were not associated with increased WMH counts.

The proportional odds for the relation between WMH and several neuropsychological measures were also significant. Worse performance on the CVLT-II total raw score, short delayed free recall, short delayed cued recall, long delayed free recall, and long delayed cued recall were more likely associated with increases in WMH number category (2% to 5%; *P*-value range = .027-.005). The odds of a participant moving from lower to higher WMHs categories were 11% higher with every 10-second increase in the time to completion of the Trail Making B time to completion (OR = 1.11, 95% CI: 1.06 to 1.16, *P*-value < .001). Performance on the Pattern Comparison task and the Picture Sequence Memory tasks was also more likely to be lower as participants move from lower to higher WMH categories (OR = 0.97, 95% CI: 0.96 to 0.98, *P*-value < .001 and OR = 0.98, 95% CI: 0.98 to 0.99, *P*-value < .001, respectively). With every 2-second increase in the time to completion of the Grooved Pegboard nondominant hand, the participants moved from a lower to higher WMHs category (OR = 1.02, 95% CI: 1.01 to 1.03, *P*-value < .001).

The odds of a participant moving from a lower to a higher WMH number category were lower (OR = 0.62, 95% CI: 0.41 to 0.92, *P*-value = .021) for those not endorsing a service-connected disability compared to those responding yes to having a service-connected disability.

## DISCUSSION

In this large cohort of Veterans and Service Members, we found that despite an absence of group difference in the presence of WMH between those with positive and negative history of TBI, only the older participants who had sustained at least 1 mTBI demonstrated a statistically significant increase in the number of WMHs at a rate greater than that explained by aging alone. Our finding complements the earlier large U.S. Service Member MRI study (*N* = 834; mean age = 34.2 years) with a wider spectrum of TBI severity who were imaged after a mean postinjury interval of 3.8 years.^7^ In that study, Riedy et al. reported that 22% of their TBI sample had several WMHs that exceeded the number expected for the age of the participant, whereas only 2.4% of the uninjured comparison group had more WMHs than expected for age. We also found that the number of WMHs increased more sharply with age in the mTBI group than in the group without a history of mTBI. Thus, we not only replicated the findings of Riedy et al.^7^ but also are the first to demonstrate that this interaction of age and TBI status was not dependent on severity of TBI, as the subjects in our study all had mTBI only.

We also analyzed the relation of WMHs to other clinical and cognitive comorbidities. One interesting finding is the relation between increased levels of IGF-1 and WMHs. The somatotopic axis (including IGF-1) appears to be affected in brain injury, and given its role in stimulating protein synthesis in neurons and glial cells, may play a role in preventing secondary injury following trauma.^18,19^ In this cohort of Veterans and Service Members, increased levels of IGF-1 significantly reduce the number of WMHs, even when controlling for age (one of the primary drivers of WMHs in this study), which may provide support for this idea. Thyroid function has also been implicated in several studies (especially in the chronic phases) as contributing to the symptom profile experiences by patients who have experienced a TBI.^20^ However, in this cohort of chronic mTBI Veterans and Service Members, there was no difference in TSH levels between the groups, and there was no observable statistically significant relation between TSH levels and WMHs in either group.

Hypertension, central obesity, insulin resistance, and atherogenic dyslipidemia form a cluster of conditions that represent a metabolic syndrome^21^ that is often associated with poor cardiovascular function.^22,23^ Several of these metabolic abnormalities have been linked to WMHs in civilian cohorts without traumatic injury^3,24,25^ and are implicated in reduced white matter integrity measured by other imaging modalities (diffusion tensor imaging).^26^ This is especially true for variables such as hypertension and obesity^27,28^ which, combined with the presence of WMHs, leads many clinicians and researchers to conclude that the WMHs are primarily vascular in origin. Interestingly, the results from the present study do not demonstrate statistically significant relationships between WMHs and measures of blood pressure (both systolic and diastolic), BMI, or weight when controlling for the effects of age. Furthermore, the 2 groups (mTBI and no mTBI history) had similar means and ranges for systolic, diastolic, and BMI variables and a marginal difference for weight (*P* = .045), with no TBI history group weighing slightly more than the mTBI group (209.3 lbs vs. 205.3 lbs).

Additionally, there is the possibility that PTSD and chronic stress in postdeployment populations increase the long-term risk for cardiovascular disease and WHMs.^29^ When considering PTSD or major depressive disorder diagnosis, neither appeared to be significantly associated with an increased risk of having higher WMH counts (see Supplementary Table S1). We did find that WMHs were associated with any service-connected disability, suggesting that these lesions may be biomarkers for reduced functional status, but additional analysis will improve our understanding of these findings. Analysis of longitudinal changes in WMHs will be forthcoming as additional follow-up imaging data become available.

As reported in the civilian literature, an increased number and volume of WMHs on repeat MRI are related to cognitive decline in persons older than 50 years.^4^ Regarding cognitive outcome measures, WMHs prevalence and/or count were associated with changes in 3 cognitive performance measures, including episodic memory (CVLT-II Total Recall and Long Delay Free Recall). However, both immediate and delayed measures of recalling words on the CVLT-II were related to WMHs, but not to mTBI. An association of WMHs with processing speed-executive function measured by Trails B completion time was found with occurrence (presence/absence) only and not with count (number of WMH). This may be a limitation of the count categories we utilized. Although the ranges were based on the expert recommendation of the neuroradiologists performing the read, these categories may not represent clinically meaningful demarcations reflective of distributions of WMHs in a more general patient population.

Our findings of greater number of WMHs in mTBI participants that increase with age at a rate that exceeds the change rates in Veterans and Service Members without a history of mTBI requires additional examination of other potential factors that may moderate or mediate this finding. For example, our study also showed increased IGF-1 levels moderated this age-by-mTBI interaction finding and may be a target for additional research. IGF-1 levels may represent a chronic biomarker following injury that can help improve our understanding of secondary mechanisms of white matter damage associated with mTBI.^19^ Mechanisms associated with the underlying neuroprotective vascular effects of IGF-1 include anti-inflammation and preservation of endothelial function. Lower IGF-1 levels have also been associated with other cardiovascular risk factors such as insulin resistance and obesity as well as poorer cognitive functioning.^30,31^

There a several limitations that should be noted. First, the fact that this study focuses on mTBI limits the generalizability beyond mTBI. Second, this study is limited to basic quantitative information and is limited to simple WMH counts. Though significant associations were still discovered using these simpler metrics, future studies would benefit from using more potentially sensitive metrics (i.e., volumetric) and qualitative features (i.e., location of WMHs) of WMHs. As such, this study remains mostly descriptive, and future studies will be needed to further elucidate clinically important information. Third, this study is limited to a single time point. Given the association of these WMHs with functional changes in neuropsychological measures observed in this study, prospective monitoring of these variables will be especially important as age continues to be an important predictor of WMHs progression regardless of mTBI history.^32,33^ In the follow-up study by Reidy et al., WMHs were found to be variable in their appearance over time and future work from this group will examine the prospective changes in WMHs.^7^ Prospective study of changes in WMHs in this cohort will also help with one main limitation of this study which is that the control (or no mTBI history) group remains much smaller than the mTBI group. Fourth, control participants can impact findings in studies,^34^ and our findings are likely impacted by the fact that our groups are similar for many of the variables of interest. Ongoing data collection and follow-up imaging in the LIMBIC-CENC study will provide an opportunity to replicate these findings and analyze the effects of both mTBI and WMHs over time within the groups. This type of data will prove critical, as the civilian literature has shown that expanded lesion volume and number of WMHs over time predict cognitive decline in a number of cohorts.^1,35–37^ As such, the current analysis and findings are tentative and help set the stage for a longitudinal analysis in a follow-up paper.

## Supplementary Material

usae336_Supp

## Data Availability

The data that support the findings in this study are publicly available through the Federal Interagency Traumatic Brain Injury Registry (FITBIR) repository.
