# Supplementary material for: White Matter Hyperintensities and Mild TBI in Post-9/11 Veterans and Service Members
Source: Mil Med. 2024 Jul 13;189(11-12):e2578–87. doi: 10.1093/milmed/usae336 (PMC11536319; doi:10.1093/milmed/usae336)
Supplement: usae336_Supp [file usae336_supp.zip › Supplemental Table 1.docx]

Supplemental Table 1. Stepwise selection results. Multivariable logistic and ordinal regression for white matter hyperintensities (WMH) presence and number of WMH, respectively.

|  | **WMH presence (yes/no)** | | **Number of WMH** | |
| --- | --- | --- | --- | --- |
| **Variables** | **OR (95% CI)** | **p-value** | **Proportional OR (95% CI)** | **p-value** |
| **mTBI - Yes** | 0.04 (0,0.41) | 0.006 | 0.08 (0.01, 0.66) | 0.018 |
| **Age at baseline (yr)** | 1.03 (1,1.06) | 0.09 | 1.03 (1.01, 1.07) | 0.046 |
| **10 × Trail Making B score** | 1.07 (1.01,1.15) | 0.040 | 46.28 (0.12,17851.85) | 0.21 |
| **Do you now smoke - 2 Some days** | 1.35 (0.61,2.93) | 0.45 | 1.34 (0.64, 2.75) | 0.44 |
| **3 Not at all** | 0.88 (0.51,1.52) | 0.63 | 0.90 (0.54, 1.52) | 0.70 |
| **Unknown** | 1.41 (0.85,2.38) | 0.19 | 1.45 (0.90, 2.37) | 0.13 |
| **1 Every day** | (Reference) | (Reference) | (Reference) | (Reference) |
| **CVLTII: Long Delay Free Recall Raw Score** | 0.97 (0.87,1.07) | 0.51 | 0.95 (0.86, 1.05) | 0.34 |
| **TSH Result: (mIU/L)** | 0.89 (0.79,1) | 0.07 | 0.90 (0.79, 0.99) | 0.06 |
| **PHQ-9: Major Depressive Disorder Criteria Met** | 1.37 (0.92,2.04) | 0.12 | 1.24 (0.86, 1.77) | 0.26 |
| **GPB Non-dominant hand time (sec)** | 1.01 (1,1.02) | 0.16 | 1.01 (1.00, 1.02) | 0.08 |
| **mTBI × Age at baseline (yr)** | 1.05 (1.01,1.09) | 0.016 | 1.04 (1.00, 1.07) | 0.052 |
| **mTBI × CVLTII: Long Delay Free Recall Raw Score** | 1.12 (1,1.25) | 0.06 | 1.10 (0.98, 1.22) | 0.09 |
